# Supplementary material for: Estimating body segment parameters from three-dimensional human body scans
Source: PLoS One. 2022 Jan 5;17(1):e0262296. doi: 10.1371/journal.pone.0262296 (PMC8730461; doi:10.1371/journal.pone.0262296)
Supplement: S1 File — (DOCX) [file pone.0262296.s001.docx]

#### **Anatomical landmarking**

##### Table S-1 : Anatomical landmarking placements and definitions

|  | **Bony Landmarks Placed on Subject** | |  | **Segment Borders** | | **Comments** |
| --- | --- | --- | --- | --- | --- | --- |
| **Body Segment** | **Front View** | **Right Side** | **Segment Name** | **Proximal or Ventral** | **Distal or Caudal** |  |
| Head + Neck | Apex of the Head | Apex of the Head | Head | Apex | C7 | Segmentation on the transverse plane at the cervical C6-C7 landmark defined in frontal plane |
|  | Tip of the Chin (at C1 level) | Right Side view along Chin line | Neck |  |  |  |
| Torso | Front at C6-C7 | At level with front | Upper Trunk | C7 | Xiphoid |  |
|  | Xiphoid  Umbilicus (L3-L4) | Xiphoid | Abdomen | Xiphoid | Umbilicus |  |
|  | Iliac Crests  Pubis |  | Pelvis | Umbilicus | Plane the through the iliospinales at an angle of 37° to the midsagittal plane ^1,2^ | See **Figure 3** in main text |
| Arm | Tip of 3^rd^ digit | Tip of 3^rd^ digit | Hand | Stylion | - |  |
|  | Center of Styloid  Center of Elbow Joint | In line with Styloid  In line with elbow joint | Forearm | Radial | Stylion |  |
|  | Acromion |  | Upper Arm | Acromion | Radial | Segmentation through the acromion by sagittal plane with arm abducted at 90 |
| Leg | At level of Greater Trochanter  Patella at level of femoral condyle | Greater Trochanter    At level with femoral condyle | Thigh | Plane the through the iliospinales at an angle of 37° to the midsagittal plane * | Tibiale | Distal cut is at approximate plane defined by Femoral condyles |
|  | At level of later malleolus | Lateral malleolus | Shank | Tibiale | Sphyrion |  |
|  | Tip of the longest toe | Tip of the longest toe | Foot | Sphyrion | - |  |

Paolo de Leva. Adjustments to Zatsiorsky-Seluyanov’s Segment Interia Parameters. *J Biomech*. 1996;29(9):1223-1230 2).Pearsall DJ, Reid JG, Livingston L a. Segmental inertial parameters of the human trunk as determined from computed tomography. *Ann Biomed Eng*. 1996;24(2):198-210. http://www.ncbi.nlm.nih.gov/pubmed/8678352
