# Supplementary material for: Estimating body segment parameters from three-dimensional human body scans
Source: PLoS One. 2022 Jan 5;17(1):e0262296. doi: 10.1371/journal.pone.0262296 (PMC8730461; doi:10.1371/journal.pone.0262296)
Supplement: S2 File — (DOCX) [file pone.0262296.s002.docx]

#### **Uniform density values**

##### Table S-2: The uniform density values for each segment that we used for our study [[1], [2]](https://paperpile.com/c/cvOHoT/wZye7+AbtsG)

| **Body Segment** | **Density (kg/m^3^ )** |
| --- | --- |
| Head | 1070 |
| Torso | 820 |
| Ab | 1010 |
| Pelvis | 1020 |
| Thigh | 1040 |
| Shank | 1080 |
| Foot | 1080 |
| Arm | 1060 |
| Forearm | 1100 |
| Hand | 1105 |
|  |  |

#### **Regression equations**

The regression equation we used in our study are based off of the study by Zatsiorsky and Seluyanov 1983.

The equations are in the form :

$$Y = B_{0}+B_{1}BW+B_{2}H$$

where $Y$is a mass inertia characteristic, $BW$ is the body weight in kg of the participant and $H$ is the height of the participant in cm. The parameters from this equation ($B_{0}B_{1}B_{2}$) can be determined using Table A-2 for males and Table A-3 for females.

##### Table S-3: Coefficients of multiple regression equations for estimating the inertial properties for male body segments from known body mass and height.

|  | **I_long_(kg cm^2^)** | | | **I_ml_ (kg cm^2^)** | | | **I_ap_ (kg cm^2^)** | | |  | | | **Segment Mass (kg)** | | |
| --- | --- | --- | --- | --- | --- | --- | --- | --- | --- | --- | --- | --- | --- | --- | --- |
| **Male** | **B0** | **B1** | **B2** | **B0** | **B1** | **B2** | **B0** | **B1** | **B2** |  |  |  | **B0** | **B1** | **B2** |
| Head | 61.6 | 1.72 | 0.0814 | -112 | 1.43 | 1.73 | -78 | 1.171 | 1.519 |  |  |  | 1.296 | 0.0171 | 0.0143 |
| Up-Trunk | 561 | 36.03 | -9.98 | 367 | 18.3 | -5.73 | 81.2 | 36.73 | -5.97 |  |  |  | 8.2144 | 0.1862 | -0.0584 |
| Abdomen | 1501 | 43.14 | -19.8 | 263 | 26.7 | -8 | 618.5 | 39.8 | -12.87 |  |  |  | 7.181 | 0.2234 | -0.0663 |
| Pelvis | -775 | 14.7 | 1.685 | -934 | 11.8 | 3.44 | -1568 | 12 | 7.741 |  |  |  | -7.498 | 0.0976 | 0.04896 |
| Thigh | -13.5 | 11.3 | -2.28 | -3690 | 32.02 | 19.24 | -3557 | 31.7 | 18.61 |  |  |  | -2.649 | 0.1463 | 0.0137 |
| Shank | -70.5 | 1.134 | 0.3 | -1152 | 4.594 | 6.815 | -1105 | 4.59 | 6.63 |  |  |  | -1.592 | 0.0362 | 0.0121 |
| Foot | -15.48 | 0.0144 | 0.088 | -97.09 | 0.414 | 0.614 | -100 | 0.48 | 0.626 |  |  |  | -0.829 | 0.0077 | 0.0073 |
| Arm | -16.9 | 0.662 | 0.0435 | -232 | 1.525 | 1.343 | -250.7 | 1.56 | 1.512 |  |  |  | 0.25 | 0.03012 | -0.0027 |
| Forearm | 5.66 | 0.306 | -0.088 | -67.9 | 0.855 | 0.376 | -64 | 0.95 | 0.34 |  |  |  | 0.3185 | 0.01445 | -0.00114 |
| Hand | -6.26 | 0.0762 | 0.0347 | -13.68 | 0.088 | 0.092 | -19.5 | 0.17 | 0.116 |  |  |  | -0.1165 | 0.0036 | 0.00175 |

##### Table A-4 : Coefficients of multiple regression equations for estimating the inertial properties for female body segments from known body mass and height

|  | **I_long_ (kg cm^2^)** | | | **I_ml_ (kg cm^2^)** | | | **I_ap_ (kg cm^2^)** | | |  | | | **Segment Mass (kg)** | | |
| --- | --- | --- | --- | --- | --- | --- | --- | --- | --- | --- | --- | --- | --- | --- | --- |
| **Female** | **B0** | **B1** | **B2** | **B0** | **B1** | **B2** | **B0** | **B1** | **B2** |  |  |  | **B0** | **B1** | **B2** |
| Head | -35.48 | 2.43 | 0.237 | 66.4 | -0.447 | 1.29 | 217.8 | -0.032 | 0.059 |  |  |  | 2.388 | -0.001 | 0.015 |
| Up-Trunk | -2823.2 | 25.8 | 12.8 | -2075 | 15.6 | 9.4 | -4038.5 | 28.6 | 20 |  |  |  | -16.593 | 0.14 | 0.0995 |
| Abdomen | -672.9 | 1.47 | 7.53 | -546 | 2.87 | 5.1 | -368.7 | -6.22 | 8.86 |  |  |  | -2.741 | 0.031 | 0.056 |
| Pelvis | -715.9 | 23.5 | -1.106 | -633 | 10.8 | 2.26 | -987.6 | 14.9 | 3.76 |  |  |  | -4.908 | 0.124 | 0.0272 |
| Thigh | 1339.8 | 6.3 | -8.28 | -2659.4 | 50.35 | 6.96 | -4033.4 | 44.99 | 17.08 |  |  |  | 5.185 | 0.183 | -0.042 |
| Shank | -53.2 | 0.284 | 0.489 | -943.3 | -2.51 | 8.47 | -963.1 | -3.57 | 9.04 |  |  |  | -0.436 | -0.011 | 0.0238 |
| Foot | 23.9 | 0.337 | -0.059 | -61.4 | 0.348 | 0.406 | -92.24 | 0.486 | 0.558 |  |  |  | -1.207 | -0.0175 | 0.0057 |
| Arm | -118.6 | 1.19 | 0.44 | -330.4 | -0.461 | 2.67 | -151.4 | 0.107 | 1.554 |  |  |  | 0.206 | 0.0053 | 0.0066 |
| Forearm | 7.4 | 0.21 | -0.08 | -138.5 | 0.533 | 0.887 | -132.1 | 0.62 | 0.825 |  |  |  | 0.295 | 0.009 | 0.0003 |
| Hand | -2.138 | 0.053 | 0.0073 | -5.79 | 0.087 | 0.034 | -5.71 | 0.122 | 0.035 |  |  |  | -0.116 | 0.0017 | 0.002 |

#### **References**

[1] [R. F. Chandler, C. E. Clauser, J. T. McConville, H. M. Reynolds, and J. W. Young, “Investigation of inertial properties of the human body,” Air Force Aerospace Medical Research Lab Wright-Patterson AFB OH, 1975. [Online]. Available:](http://paperpile.com/b/cvOHoT/wZye7) <https://apps.dtic.mil/sti/citations/ADA016485>[.](http://paperpile.com/b/cvOHoT/wZye7)

[2] [D. J. Pearsall, J. G. Reid, and L. A. Livingston, “Segmental inertial parameters of the human trunk as determined from computed tomography,” *Ann. Biomed. Eng.*, vol. 24, no. 2, pp. 198–210, Mar. 1996.](http://paperpile.com/b/cvOHoT/AbtsG)
