# Supplementary material for: Estimating body segment parameters from three-dimensional human body scans
Source: PLoS One. 2022 Jan 5;17(1):e0262296. doi: 10.1371/journal.pone.0262296 (PMC8730461; doi:10.1371/journal.pone.0262296)
Supplement: S3 File — (DOCX) [file pone.0262296.s003.docx]

#### **Proximal center of mass definitions**


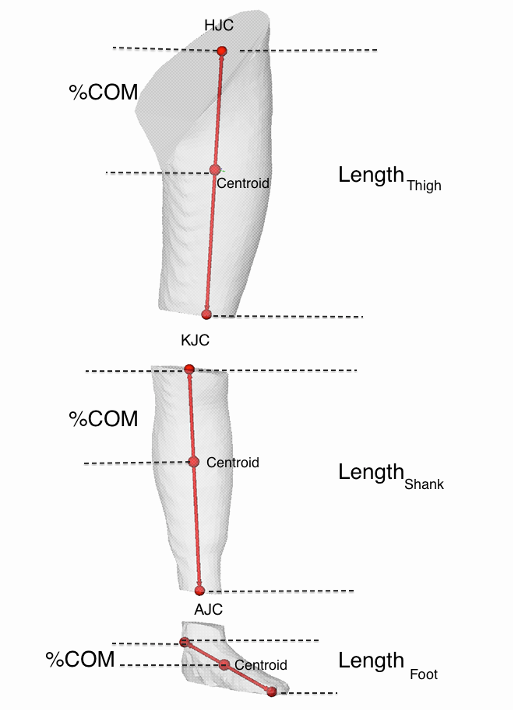


##### Figure A-2: Leg body segments showing centre of mass (Centroid), proximal distance of COM as a percentage of the total longitudinal length of the segment (%COM) and longitudinal length of segment (Length). Estimated Hip joint center (HJC), Knee joint center (KJC), and Ankle joint center (AJC) are shown.

#####
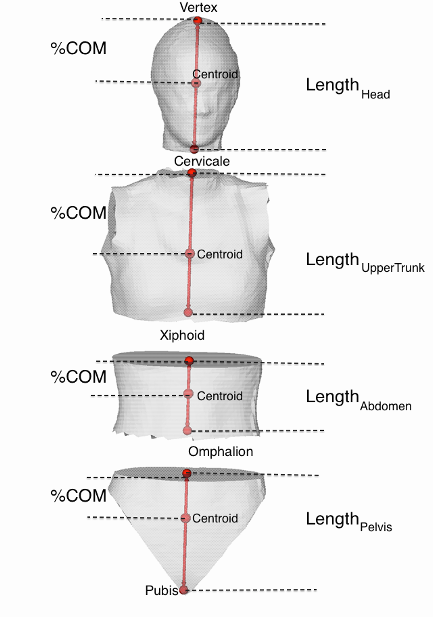


##### Figure A-3: Upper body segments showing centre of mass (Centroid), proximal distance of COM as a percentage of the total longitudinal length of the segment (%COM) and longitudinal length of segment (Length). Estimated Vertex, Cervicale, Xiphoid, Omphalion and Pubis are also shown.

#### **
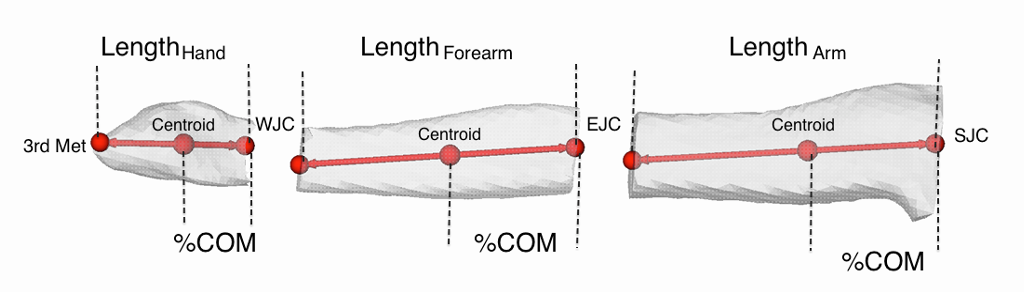
**

##### Figure A-4: Arm segmentation showing centre of mass (Centroid), proximal distance of COM as a percentage of the total longitudinal length of the segment (%COM) and longitudinal length of segment (Length). Estimates of the third metatarsal (3^rd^ Met), Wrist joint center (WJC), Elbow joint centre (EJC), and Shoulder joint center (SJC) define the estimated endpoints of each segment.
