# Supplementary material for: Estimating body segment parameters from three-dimensional human body scans
Source: PLoS One. 2022 Jan 5;17(1):e0262296. doi: 10.1371/journal.pone.0262296 (PMC8730461; doi:10.1371/journal.pone.0262296)
Supplement: S4 File — (DOCX) [file pone.0262296.s004.docx]

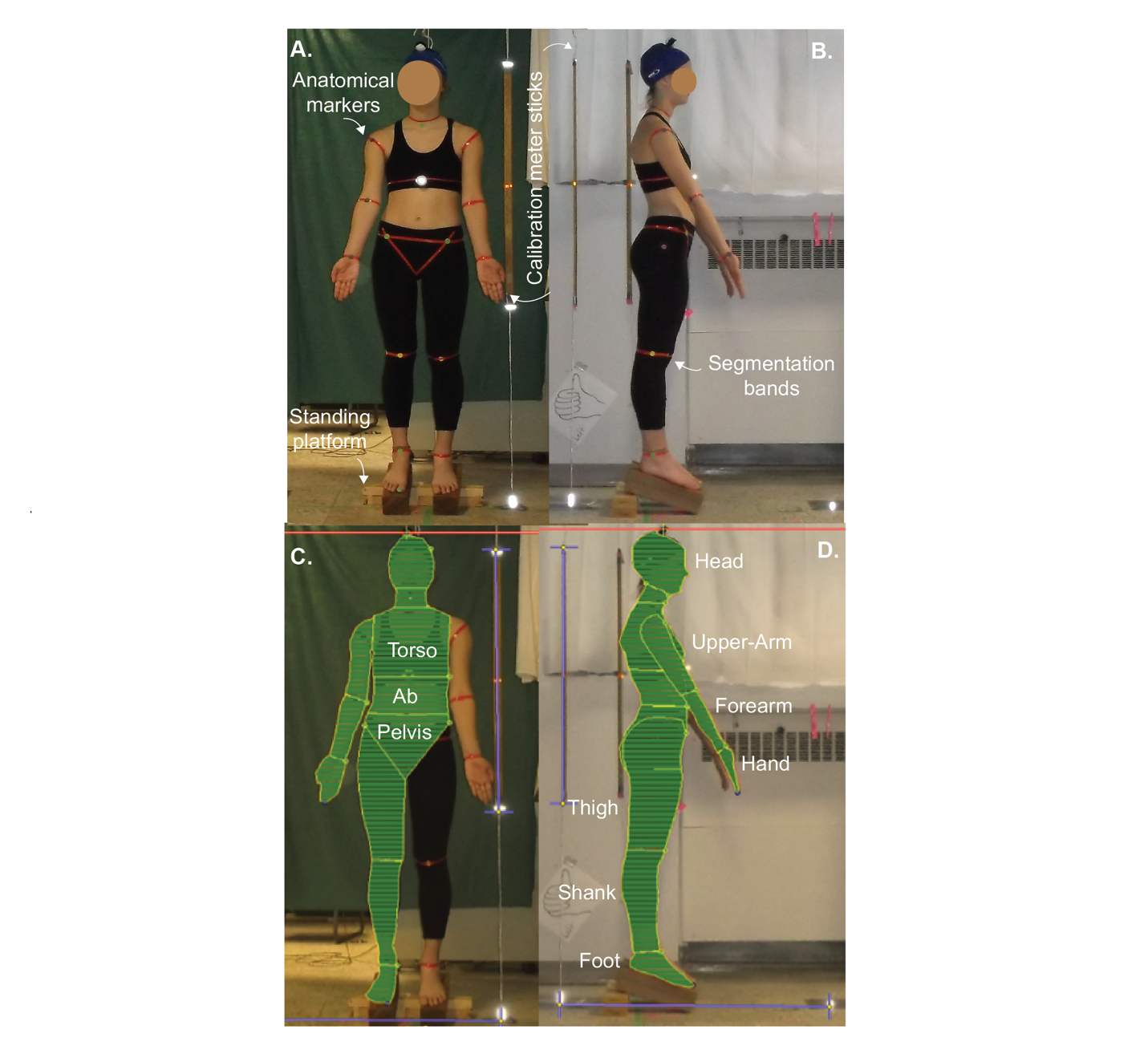


**Figure S-2:** Photographic method used for estimating participant-specific BSPs. **A.** frontal view, **B.** sagittal view, **C.** digitized frontal view showing segmentation boundaries, and D. digitized sagittal view showing segmentation boundaries.
