# Supplementary material for: Estimating body segment parameters from three-dimensional human body scans
Source: PLoS One. 2022 Jan 5;17(1):e0262296. doi: 10.1371/journal.pone.0262296 (PMC8730461; doi:10.1371/journal.pone.0262296)
Supplement: S5 File — (DOCX) [file pone.0262296.s005.docx]

#### **Measuring Cylinder**


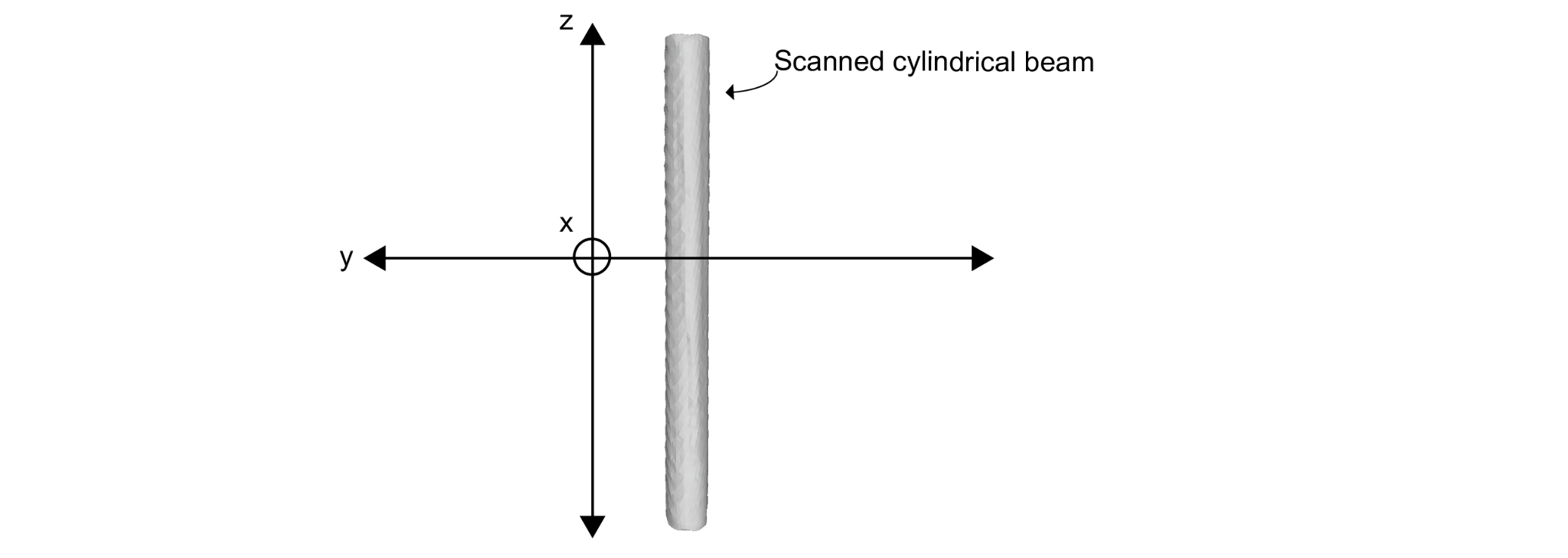


##### Figure S-1: Example 3D scanned cylindrical beam. Coordinate system shown for reference.

A cylinder was measured 25 times and determined to have a radius of 4.1±0.1cm a height of 94.0±0.1cm and a volume of 4964.2±242.0 cm^3.^ Using these values and the equation for the mass moments of inertia of a cylinder the interior parameters of the cylinder were determined using a measured method.

$$Ixx = 1/12mh^{2}+1/4 mr^{2}$$

$$Iyy = 1/12mh^{2}+1/4 mr^{2}$$

$$Izz = 1/2 mr^{2}$$

$$Volume = \pi r^{2}h$$

Mass moments of inertia along a principal axis of a cylinder with radius r and height h. The mass was calculated based on a cylindrical density of 0.001kg/cm^3^

##### Table S-5: Comparison between measured and experimentally obtained parameters for a cylindrical tube or radius 4.1±0.1cm and length 97±0.1 cm.

|  | **Measured** | | | **Estimated using Kinect 3D scanning protocol** | | | | | |  |  |
| --- | --- | --- | --- | --- | --- | --- | --- | --- | --- | --- | --- |
| **Parameter** | **Value** | | | **Value** | | | **Max** | **Min** | **Range** | **Mean difference** | **% difference** |
| Volume [cm^3^] | 4964 | ± | 242 | 5173 | ± | 204 | 4927 | 5550 | 623 | 209 cm^3^ | 4.12 |
| Length [cm] | 94.1 | ± | 0.1 | 93.6 | ± | 2 | 97.5 | 89.8 | 7.7 | -0.5 cm | -0.53 |
| pCOM [%] | 50% | ± | # | 49.8% | ± | 0.9 | 52.3% | 48.2% | 4.1% | -0.2% | -0.40 |
| Ixx [kgcm^2^] | 3676 | ± | 178 | 3707 | ± | 197 | 4049 | 3449 | 600 | 31 kgcm^2^ | 0.84 |
| Iyy [kgcm^2^] | 3676 | ± | 178 | 3702 | ± | 196 | 4043 | 3445 | 598 | 26 kgcm^2^ | 0.70 |
| Izz [kgcm^2^] | 42 | ± | 2 | 45.3 | ± | 4.4 | 57.9 | 39.0 | 188 | 3.3 kgcm^2^ | 7.56 |
