# Supplementary material for: Estimating body segment parameters from three-dimensional human body scans
Source: PLoS One. 2022 Jan 5;17(1):e0262296. doi: 10.1371/journal.pone.0262296 (PMC8730461; doi:10.1371/journal.pone.0262296)
Supplement: S6 File — (DOCX) [file pone.0262296.s006.docx]

#### **Total body volume estimations between repeated scans**

##### Table A-6: The total body volume (cm^3^) from 3 repeated scans (A, B, and C) estimated using the proposed 3D scanning approach for both males and females. The within-participant standard deviation (SD) and coefficients of variations (CV) are shown. We found no statistical differences for the total volume of repeated scans for both males (p =0.12) and females (p=0.22).

|  | **Male (n=10)** | | | | |  | **Female (n=11)** | | | | |
| --- | --- | --- | --- | --- | --- | --- | --- | --- | --- | --- | --- |
| Participant | Scan A  (cm^2^) | Scan B (cm^2^) | Scan C (cm^2^) | SD (cm^2^) | CV(%) |  | Scan A (cm^2^) | Scan B (cm^2^) | Scan C (cm^2^) | SD (cm^2^) | CV(%) |
| 1 | 741.8 | 707.6 | 710.8 | 18.9 | 2.6 |  | 829.2 | 825.0 | 815.6 | 7.0 | 0.8 |
| 2 | 759.3 | 732.8 | 750.2 | 13.5 | 1.8 |  | 840.1 | 826.0 | 819.9 | 10.4 | 1.3 |
| 3 | 835.2 | 837.1 | 776.1 | 34.7 | 4.3 |  | 652.9 | 665.5 | 682.0 | 14.6 | 2.2 |
| 4 | 713.5 | 726.7 | 740.9 | 13.7 | 1.9 |  | 728.8 | 697.0 | 715.5 | 15.9 | 2.2 |
| 5 | 722.9 | 712.5 | 703.4 | 9.8 | 1.4 |  | 806.8 | 801.1 | 806.6 | 3.3 | 0.4 |
| 6 | 770.6 | 747.4 | 746.7 | 13.6 | 1.8 |  | 729.0 | 720.7 | 714.7 | 7.2 | 1.0 |
| 7 | 802.1 | 807.3 | 781.2 | 13.8 | 1.7 |  | 637.2 | 626.2 | 611.9 | 12.7 | 2.0 |
| 8 | 726.6 | 708.1 | 721.8 | 9.6 | 1.3 |  | 727.1 | 734.8 | 765.2 | 20.1 | 2.7 |
| 9 | 785.2 | 776.1 | 785.8 | 5.4 | 0.7 |  | 857.2 | 825.3 | 820.9 | 19.8 | 2.4 |
| 10 | 665.1 | 662.7 | 665.1 | 1.4 | 0.2 |  | 627.5 | 604.1 | 621.5 | 12.2 | 2.0 |
| 11 |  |  |  |  |  |  | 666.2 | 671.3 | 672.9 | 3.5 | 0.5 |

#### **Mean ICC estimates across repeated segmentations for all body segment parameters**

|  | **Male (n=10)** | | | | | | **Female (n=11)** | | | | | |
| --- | --- | --- | --- | --- | --- | --- | --- | --- | --- | --- | --- | --- |
| **Segment** | **Volume** | **Ixx** | **Iyy** | **Izz** | **Length** | **pCOM** | **Volume** | **Ixx** | **Iyy** | **Izz** | **Length** | **pCOM** |
| **Head** | 0.94 | 0.90 | 0.90 | 0.95 | 0.78 | 0.91 | 0.91 | 0.88 | 0.89 | 0.92 | 0.80 | 0.73 |
| **Torso** | 0.95 | 0.94 | 0.93 | 0.97 | 0.81 | 0.56 | 0.98 | 0.97 | 0.97 | 0.99 | 0.92 | 0.78 |
| **Ab** | 0.98 | 0.97 | 0.97 | 0.98 | 0.97 | 0.92 | 0.99 | 0.99 | 0.99 | 0.99 | 0.96 | 0.71 |
| **Pelvis** | 0.91 | 0.90 | 0.94 | 0.94 | 0.72 | 0.75 | 0.89 | 0.83 | 0.92 | 0.93 | 0.53 | 0.68 |
| **Thigh** | 0.86 | 0.83 | 0.83 | 0.85 | 0.83 | 0.89 | 0.97 | 0.95 | 0.95 | 0.97 | 0.86 | 0.87 |
| **Shank** | 0.90 | 0.93 | 0.93 | 0.88 | 0.95 | 0.96 | 0.97 | 0.98 | 0.98 | 0.96 | 0.99 | 0.77 |
| **Foot** | 0.64 | 0.69 | 0.57 | 0.71 | 0.40 | 0.48 | 0.93 | 0.96 | 0.95 | 0.92 | 0.80 | 0.68 |
| **Arm** | 0.70 | 0.47 | 0.53 | 0.75 | 0.90 | 0.78 | 0.84 | 0.86 | 0.85 | 0.88 | 0.91 | 0.31 |
| **Forearm** | 0.92 | 0.84 | 0.86 | 0.96 | 0.62 | 0.89 | 0.75 | 0.84 | 0.82 | 0.81 | 0.93 | 0.76 |
| **Hand** | 0.94 | 0.91 | 0.90 | 0.95 | 0.78 | 0.26 | 0.36 | 0.64 | 0.67 | 0.50 | 0.77 | 0.82 |
| **Full Body** | 0.96 |  |  |  |  |  | 0.99 |  |  |  |  |  |
